# Supplementary material for: Biological batch normalisation: How intrinsic plasticity improves learning in deep neural networks
Source: PLoS One. 2020 Sep 23;15(9):e0238454. doi: 10.1371/journal.pone.0238454 (PMC7511202; doi:10.1371/journal.pone.0238454)
Supplement: Attachment — Submitted filename: PLoSOne_Responses.pdf [file pone.0238454.s001.pdf]

Response to Reviewers' Comments for PONE-D-19-29067

We would like to thank the editors and reviewers for their valuable efforts on this paper. We have read the comments carefully and revised the manuscript accordingly. We hope that the improvements and changes in the revised manuscript, as well as our replies to the reviewers, will be satisfactory.

In any case, we are sincerely grateful for your perspective and valuable suggestions.

To summarise the changes, we have identified an error in the rule we first presented and fixed this error. As a result, the two separate tracks of experiments for the two separate rules are now unified into a single section. We have updated the rest of the paper to reflect these changes. Our hope is that the reduced length and single set of experiments will improve readability and the central thesis of our work—that intrinsic plasticity can be used to improve learning by solving the vanishing gradient problem in deep networks.

Reviewer 1

Comment 1: The authors investigate two local learning rules applied to shallow and deep neural network learning processes. The learning dynamics is shown to be more stable for a wider range of learning rates. Adding the weight decay rule even makes the network converge to better solutions. These conclusions are supported with two example data sets and some analytical analysis of how the learning rules enhance the learning dynamics.

Comment 2: Although the manuscript contains some insights, the results are mixed and not convincingly presented. Biological plausibility of the local learning rule is a plus, but it should not be the primary emphasis for the additional learning rules, especially since the main learning process is back propagation. Artificial neural networks (ANNs) are at best remotely related to biology. Its main strength is in its performance. As such, biological plausibility added on to ANNs should aim to improve the performance.

Reply to the comment:
There are newer recurrent architectures that implement backprop-like learning, such as [Guergiuev et al., 2017, Spratling, 2008, Lillicrap et al., 2016, Bogacz, 2017]. These methods are biologically plausible and have been shown to approximate backpropagation. Hence, they are capable of achieving results comparable to state of the art ANNs. Furthermore, with the advent of neuromorphic hardware approaching, local learning rules that address the vanishing gradient problem are an important direction of research. The biological plausibility of our method is not only important for bridging the gap in theory between machine learning and biology, but also for providing a method that improves learning on hardware that we are currently unable to test on, particularly in an online and distributed environment. We have added to the final paragraph in the biological plausibility subsection that speaks to this importance.

Nevertheless, we hope that our revised manuscript will assuage the reviewer's concern regarding

the gap between performance and theory. We've identified the error in both methods previously used, and now present a single IP method that is both theoretically sound and demonstrates improved performance across all tests.

Comment 3: The improvements over regular ANNs are seem in the weight decay rule, but the authors also point out theoretical difficulty related to it. The other rule performs even worse than regular ANNs. These make one wonder what we have learned in this work. The manuscript is not well written. At the end the authors even mention "this thesis", which is a reflection of poor quality control.

Reply to the comment:
Good catch. This work does indeed stem from a thesis published in 2019. However, the work has been extended and improved upon.

Comment 4: The authors might consider considerably improve the manuscript by emphasizing on some variant of the weight decay rule and convincingly make the case that such an addition is useful for stability of learning and also improves accuracy.

Reply to the comment:
We appreciate the reviewer's concerns, and agree that the two methods proposed in the first draft were somewhat confusing, and the mixed results failed to demonstrate the value of intrinsic plasticity in a compelling manner. We started this line of research with an intuition that an unintended benefit of information maximisation is its normalising behaviour and that centering neuronal activities over their input distributions could solve the vanishing gradient problem. We wrongly computed the steady-state solutions for the gain, and treated it as an integrator. We have since identified this flaw and the rule presented in this draft is exactly the variant we hope you are looking for. As stated above, it does not have the theoretical flaw of being unable to converge to biases outside of $[-1,1]$ and its performance is in line with what we originally expected to find.

In short, the results presented here merge the two methods previously presented and benefit from being both a stronger theoretical foundation as well as improved performance.

Reviewer 2

Comment 5: The paper is well orgnized and easy to read. The intrinsic plasticity is found to be helpful in deep learning. The results are nice, but some related works on intrinsic plasticity in neural networks should be involved, see e.g. [R1-R3]. I recommend to accept this paper once the comments have been addressed.

[R1] Self-orgnized Spiking nueral P Systems

[R2] Axon neural systems

[R3] Spiking neural P systems with plastic synapes

Reply to the comment:
We thank the reviewer for their time and positive comments. We have looked at the suggested papers, and while the work is interesting, we do not feel that they are directly relevant to the topic in this manuscript. More precisely, our impression of these works is that they are primarily concerned with the mechanisms that underlie spike-timing-dependent plasticity and membrane computing can be used to model non-synaptic phenomena in neurons. While membrane computing is expressive enough to describe intrinsic plasticity, the aim of our work is to abstract the various forms of intrinsic plasticity, such as short-term adaptivity and long-term habituation, to focus on how it impacts the dynamics of synaptic updates.

We hope that this response is satisfactory. If the reviewer feels that we have missed an important relationship with that work, then we would be happy to address specific comments.

References

Bogacz, R. (2017). A tutorial on the free-energy framework for modelling perception and learning. *Journal of Mathematical Psychology*, 76:198–211.

Guergiuev, J., Lillicrap, T. P., and Richards, B. A. (2017). Towards deep learning with segregated dendrites. *eLife*, (6:e22901).

Lillicrap, T. P., Cownden, D., Tweed, D. B., and Akerman, C. J. (2016). Random feedback weights support learning in deep neural networks. *Nature Communications*, 7:13276.

Spratling, M. (2008). Predictive coding as a model of biased competition in visual attention. *Vision Research*, 48(12):1391–1408.
